# Excess risk of male breast cancer in the Norwegian Offshore Petroleum Workers (NOPW) cohort: a possible link to extreme night shift work?

**DOI:** 10.1186/s13058-021-01485-w

**Published:** 2021-11-18

**Authors:** Fei-Chih Liu, Marit B. Veierød, Kristina Kjærheim, Trude E. Robsahm, Reza Ghiasvand, Ronnie Babigumira, Nita K. Shala, Leon A. M. Berge, Giske Ursin, Tom K. Grimsrud, Jo S. Stenehjem

**Affiliations:** 1 Department of Research, Cancer Registry of Norway, Majorstuen, P.O. Box 5313, 0304 Oslo, Norway; 2 Department of Biostatistics, Oslo Centre for Biostatistics and Epidemiology, Institute of Basic Medical Sciences, University of Oslo, Oslo, Norway; 3 Oslo Centre for Biostatistics and Epidemiology, Oslo University Hospital, Oslo, Norway; 4 Cancer Registry of Norway, Oslo, Norway; 5 Department of Nutrition, Institute of Basic Medical Sciences, University of Oslo, Oslo, Norway; 6 Department of Preventive Medicine, University of Southern California, Los Angeles, CA USA

**Keywords:** Night shift work, Male breast cancer, Petroleum workers, Cohort

**LETTER-TO-THE–EDITOR**

Male breast cancer (MBC) is a rare disease with some recognised risk factors, such as age, genetic disorders, family history, hormonal influence and radiation [1, 2]. Night shift work (NSW) disrupts the circadian rhythm and may lead to sleep disturbances and changes in sex hormone levels [3], which has been suggested to increase breast cancer risk in female nurses [4]. There is, however, a lack of knowledge regarding the relationship between NSW and MBC.

The Norwegian Offshore Petroleum Workers (NOPW) cohort was established in 1998 by recruiting 25,347 male workers who answered a questionnaire. Each such worker had at least one employment in oil and gas operations on the Norwegian continental shelf 1965–1998. All workers experienced a schedule with 12-h workdays for two weeks; consisting of either day work only, seven day and seven night shifts, or night shifts only; followed by a four-week off-duty period. Ever exposure to NSW was defined as having ever worked night shift during employment. We previously reported a twofold excess MBC incidence in the NOPW cohort compared to the general Norwegian population (standardized incidence ratio [SIR] = 2.18, 95% confidence interval [CI] 1.13–3.81) [5]. By December 31st, 2019, the number of prospectively recorded MBC cases had increased from 12 to 14, giving SIRs of 2.10 (95% CI 1.15–3.53) overall, 2.49 (1.14–4.73) in ever exposed, and 1.77 (0.57–4.12) in those never exposed to NSW (Table 1). We conducted internal comparisons, using a Cox proportional hazards model adjusted for age (timescale) and education. Males ever exposed to NSW were at 33% increased risk compared to those never exposed, although the estimate was not statistically significant (Table 1). Analyses were performed with Stata version 17 (StataCorp, College Station, TX, USA).

**Table 1:** Male breast cancer risk by night shift work in the Norwegian Offshore Petroleum Workers cohort

	Obs	Exp	SIR (95% CI)^a^
*SIR analysis*			
Overall	14	6.66	2.10 (1.15–3.53)
Night shift work			
Never	5	2.83	1.77 (0.57–4.12)
Ever	9	3.61	2.49 (1.14–4.73)
Missing	0	0.22	-

	Complete case (n = 24,438)			Multiple imputation^c^ (n = 25,284; 14 cases)
	No. of participants	No. of cases	HR (95% CI)	HR (95% CI)
*Cox regression analysis*^b^				
Night shift work				
Never	10,489	5	1.00 (reference)	1.00 (reference)
Ever	13,949	8	1.18 (0.38, 3.63)	1.33 (0.44, 3.98)

Follow-up period was from 1st July 1999 to 31st December 2019

Analyses were performed using Stata 17 (StataCorp, TX, USA)

*Obs.* observed, *CI* confidence interval, *Exp.* expected, *No.* number, *HR* hazard ratio, *SIR* standardized incidence ratio

^a^SIRs calculated from sex-, age- (5-year) and time- (1-year) specific incidence

rates for the Norwegian population, assuming a Poisson distribution of the observed cases

^b^Cox proportional hazards model adjusted for age (timescale) and education

^c^3% of the cohort had missing information on education and night shift work, and 5 datasets were imputed

We report this apparent occupational risk of MBC to inspire further research. In our cohort, cancers were prospectively recorded, and analyses were controlled for age and education. In separate analyses, we explored the association with exposure to benzene and chlorinated degreasers and found no sign of increased MBC risk. Lifestyle factors could possibly contribute to breast cancer risk among shift workers, though some of the effect would be adjusted for by education as a proxy variable. With these reservations, our data suggest that extreme NSW may contribute to MBC risk.

## Data Availability

The data that support the findings of this study are available from the CRN (cohort data and cancer data) and the National Population Register (death and emigration data) but restrictions apply to the availability of these data, which were used under license for the current study, and so are not publicly available. Requests for data sharing/case pooling for projects with necessary approvals and legal basis according to the EU General Data Protection Regulation (GDPR) may be directed to principal investigator Dr. Tom K. Grimsrud; email: tom.k.grimsrud@kreftregisteret.no.
